# Internal Mammary Sentinel Lymph Node Biopsy in Clinically Axillary Lymph Node-Positive Breast Cancer: Diagnosis and Implications for Patient Management

**DOI:** 10.1245/s10434-019-07705-0

**Published:** 2019-08-12

**Authors:** Peng-Fei Qiu, Rong-Rong Zhao, Wei Wang, Xiao Sun, Peng Chen, Yan-Bing Liu, Zhi-Guo Liu, Yong-Sheng Wang

**Affiliations:** 1 Breast Cancer Center, Shandong Cancer Hospital Affiliated to Shandong University, Shandong Cancer Hospital and Institute, Shandong First Medical University and Shandong Academy of Medical Sciences, Jinan, Shandong China; 2 Department of Medicine, Shandong Cancer Hospital Affiliated to Shandong University, Shandong Cancer Hospital and Institute, Shandong First Medical University and Shandong Academy of Medical Sciences, Jinan, Shandong China; 3 Department of Radiotherapy, Shandong Cancer Hospital Affiliated to Shandong University, Shandong Cancer Hospital and Institute, Shandong First Medical University and Shandong Academy of Medical Sciences, Jinan, Shandong China; 4 Department of Nuclear Medicine, Shandong Cancer Hospital Affiliated to Shandong University, Shandong Cancer Hospital and Institute, Shandong First Medical University and Shandong Academy of Medical Sciences, Jinan, Shandong China

## Abstract

**Background:**

Routine performance of internal mammary sentinel lymph node biopsy (IM-SLNB) remains a subject of debate due to no clinical relevance in breast cancer, because it was performed only in clinically axillary lymph node (ALN)-negative patients. In this study, IM-SLNB was performed in clinically ALN-positive patients, and its impact on nodal staging and therapeutic strategy were subsequently analyzed.

**Methods:**

Clinically ALN-positive patients who underwent IM-SLNB were enrolled in this prospective study. Statistical analysis was performed using Chi square test, Mann–Whitney *U* and logistic regression models with a significance level of 0.05.

**Results:**

Among the 352 recruited patients, the internal mammary sentinel lymph node (IMSLN) visualization rate of patients who received initial surgery and neoadjuvant systemic therapy (NST) was 71.9% (123/171) and 33.1% (60/181), respectively. The 183 patients who underwent IM-SLNB successfully had the average time duration of 7 min and the median IMSLN number of 2. There were 87 positive IMSLNs in all the 347 removed IMSLNs, which were mainly concentrated in the second (50.6%) and third (34.5%) intercostal space. The IMSLN metastasis rate was 39.8% (initial surgery) and 13.3% (NST), respectively. All of the 183 IM-SLNB patients received more accurate nodal staging, 57 of whom had stage elevated, which might have prompted modifications to the therapeutic strategy.

**Conclusions:**

IM-SLNB should be routinely performed in clinically ALN-positive patients, and thus more accurate nodal staging and perfect pathologic complete response definition could be put forward. The identification of IMLN metastases by IM-SLNB might potentially influence therapeutic strategies.

As a first-echelon nodal drainage site in breast cancer, the status of axillary lymph nodes (ALN) and internal mammary lymph nodes (IMLN) is valuable both for regional staging and treatment choice.^1^,^2^ The status of ALN has been well assessed with the axillary sentinel lymph node biopsy (A-SLNB) procedure in patients with early breast cancer.^3^ For the patients receiving neoadjuvant systemic therapy (NST), the nodal pathologic complete response (pCR) was identified as no existence of metastatic carcinoma in ALN and has been shown to be associated with improved survival outcomes.^4^ However, accurate regional staging and nodal pCR definition could not be achieved by depending on the status of the ALN alone, which might lead to understage and under-/overtreatment. The internal mammary sentinel lymph node biopsy (IM-SLNB) provided a less invasive method for assessing IMLN than surgical dissection and may affect decision-making for regional and systemic therapy. However, routine performance of the IM-SLNB remains a subject of debate due to the ambiguous clinical relevance.^5^

To our acknowledge, the IM-SLNB indications have not been standardized in current guidelines. Clinical work/study still referring to the indications of A-SLNB and only performing in clinically ALN-negative patients without NST, which led to the low IMLN metastasis rate (8–15%) and little influence on treatment strategy.^6^ Previous studies of extended radical mastectomy showed that the IMLN metastasis rate was 28–52% in ALN-positive patients, whereas the metastasis rate was only 5–17% in ALN-negative patients.^7^,^8^ Therefore, adjustment of regional staging, nodal pCR definition, and treatment strategy might be frequent in clinically ALN-positive patients, and these patients might really benefit from the IM-SLNB. In this prospective study, the incidence of internal mammary sentinel lymph node (IMSLN) metastasis were analyzed, and the impact of IM-SLNB on regional staging, nodal pCR definition, systemic, and locoregional treatment strategies were evaluated in clinically ALN-positive patients.

## Methods

The single-institution, prospective study was designed to determine the clinical relevance for IM-SLNB performed in clinically ALN-positive patients. The protocol and consent forms were approved by our Institutional Ethics Committee (No. SDTHEC20110324).

### Eligibility Criteria

Between February 2014 and July 2018, 352 patients with clinical stage II to III (T1-4, N1-3, M0), biopsy-proven primary breast cancer were enrolled in this study. Clinically ALN-positive was identified as that clinical physical examination and/or imaging showed abnormal ALN, and needle aspiration cytology confirmed metastasis before treatment. Exclusion criteria included metastases in clinically detected IMLN (imaging showed abnormal and fine-needle aspiration–confirmed metastasis), distant metastatic disease, and inflammatory carcinoma.

### Tracer Injection and Lymphatic Imaging

The ^99m^Tc-labeled sulfur colloid (^99m^Tc-SC) was prepared by the Nuclear Medicine Department and Sulfur colloid was labeled with ^99m^Tc after filtering through a Millipore filter with a pore size of 220  nm. In each patient, ^99m^Tc-SC (1.0–1.2 ml/22.2–55.5 MBq) was injected into the mammary gland at 6 and 12 o’clock of the areola surrounding area with the guidance of ultrasound (modified injection technique: periareolar intraparenchymal, high volume, and ultrasound guidance) 3–18 h before surgery.^9^ SPECT/CT lymphoscintigraphy was performed 30 min before surgery, and the focal accumulations of radioactivity (hotspots) outside the injection sites were identified as SLN.

### IM-SLNB Technique and Surgical Procedure

**Fig. 1:**
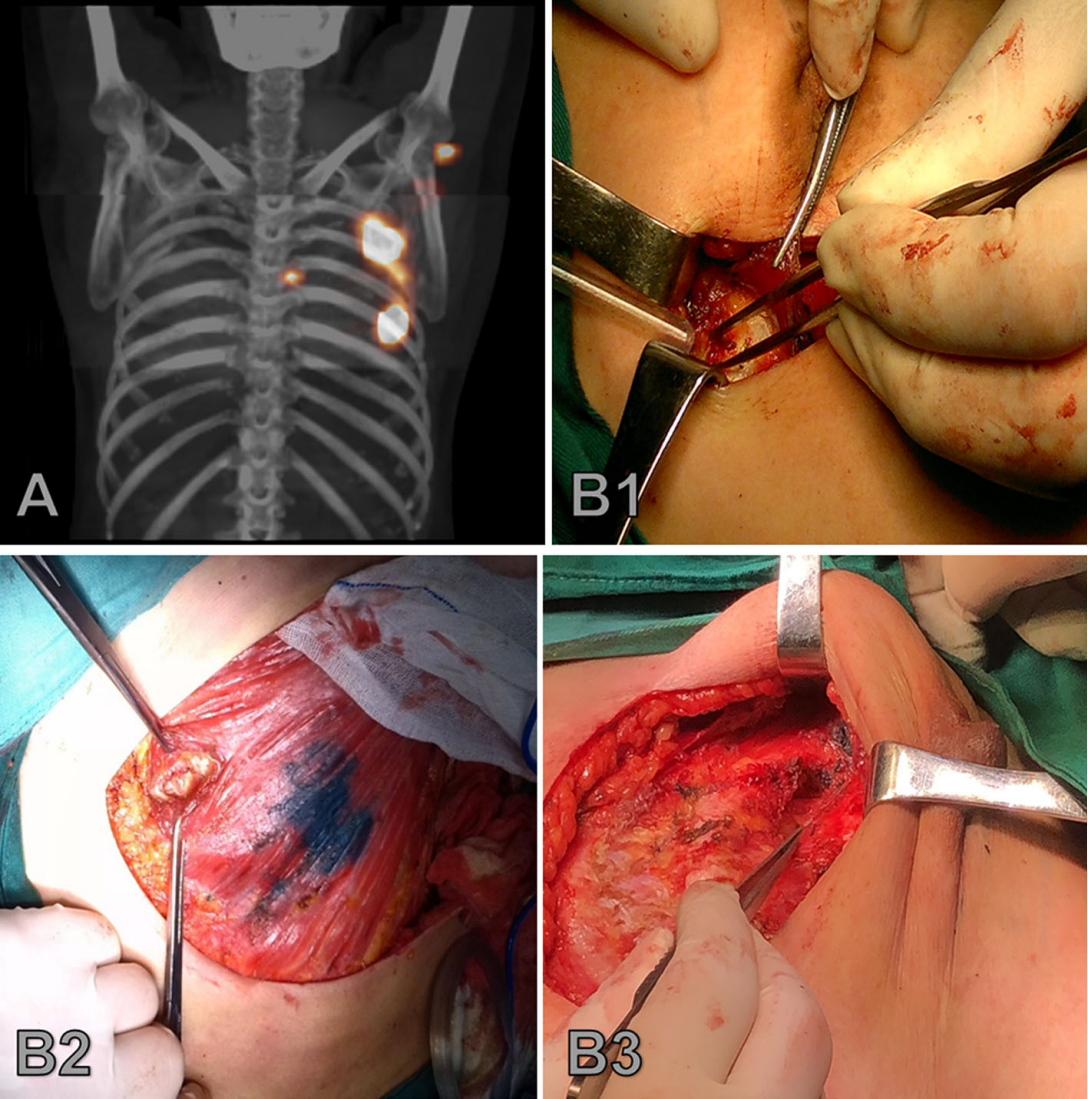
SPECT/CT imaging (**A**) and IM-SLNB procedure (**B1** Lumpectomy; **B2** Mastectomy; **B3** Reconstruction)

The IM-SLNB was usually performed after breast and axilla surgery with no additional damage to skin or ribs (if the patient underwent breast-conserving surgery and the tumor was located in the lateral, an additional 3-cm skin incision over the hotspot was required). Intraoperative identification of the IMSLN was based on gamma probe detection (Neoprobe, Neo2000 gamma detection system, Johnson & Johnson), all hotspots in the internal mammary basin were harvested (Fig. 1). Postoperative chest x-ray was performed in case of intraoperative pleural injury.

### Pathologic Evaluation

IMSLN was divided into 2-mm tissue fragments according to the long axis (short diameter < 2 mm, no division), and every tissue fragment required one layer of routine H&E staining pathological examination. Further IHC staining of CK-19 was required if H&E staining proved negative. A lymph node that was found to have any tumor in itself, including micrometastases and isolated tumor cells, was defined as positive. The nodal pathological response after NST was evaluated according to Sataloff scale (N–A: evidence of therapeutic effect, no metastatic disease. N–B: no nodal metastasis or therapeutic effect. N–C: evidence of therapeutic effect, but nodal metastasis still present. N–D: viable metastatic disease, no therapeutic effect).

### Adjuvant Therapy

Treatment strategies were decided by doctor according to the latest National Comprehensive Cancer Network (NCCN) Breast Cancer Clinical Practice Guidelines or St. Gallen Consensus, and patients participated in the decision-making. All NST patients received full course of chemotherapy regimens before surgery.

### Endpoint and Sample Size

The primary endpoint of this prospective study was the impact of IM-SLNB on regional staging (IMSLN metastasis rate) in clinically ALN-positive patients. Results of previous extended radical mastectomy studies showed that approximately 25% of IMLN were metastatic in ALN-positive patients.^1^,^7^,^8^,^10^ It was assumed that the visualization rate of IMSLN was 50% and the success rate of IM-SLNB was 90% according to our previous study.^9^ In order to reach an improvement rate at least 25% in regional staging, at the 5% significance level, a minimum of 160 patients per group were required to reach a power of 90% (two-sided test).

### Statistical Analysis

The data were analyzed with the SPSS 26.0 software. Mann–Whitney *U* or *t* test was used to compare the means of the continuous variables. Chi square test or Fisher test was used to compare the rates among the continuous variables. Univariate logistic regression analysis was used to assess the strength of the association between predictive variable and the status of IMSLN, and multivariate logistic regression analysis was applied to identify independent effects of these univariate predictive variables (Take *α* = 0.05, *P* < 0.05).

## Results

A total of 352 clinically ALN-positive patients were enrolled in this prospective study, among whom 171 received initial surgery and 181 received NST according to disease stage and patients’ preference. The clinicopathologic characteristics of these enrolled patients are presented in Table 1.

**Table 1:** Descriptive characteristics of eligible patients

Variables	Eligible patients *N *= 352		Initial surgery *N *= 171		NST *N *= 181	
	No.	%	No.	%	No.	%
Age (yr)						
Median	49		49		49	
Range	25–72		33–72		25–70	
BMI						
Median	24.8		24.6		26.0	
Range	18.0–38.1		18.0–38.1		18.4–32.4	
Clinical T stage						
T1	76	21.6	62	36.3	14	7.7
T2	186	52.8	98	57.3	88	48.6
T3	50	14.2	11	6.4	39	21.5
T4	40	11.4	0	0	40	22.1
Clinical N stage						
N1	203	57.7	118	69.0	85	47.0
N2	108	30.7	53	31.0	55	30.4
N3	41	11.6	0	0	41	22.6
Tumor location						
UOQ	179	50.9	90	52.6	89	49.2
LOQ	39	11.1	21	12.3	18	9.9
UIQ	61	17.3	32	18.7	29	16.0
LIQ	25	7.1	12	7.0	13	7.2
Central	48	13.6	16	9.4	32	17.7
Pathological type						
Ductal	329	93.5	163	95.3	166	91.7
Lobular	6	1.7	2	1.2	4	2.2
Micropapillary	17	4.8	6	3.5	11	6.1
Grade						
I	16	4.9	9	5.5	7	4.2
II	189	57.4	89	54.6	97	58.4
III	139	44.2	65	39.9	62	37.3
Tumor subtype						
HR +/HER2–	188	53.4	101	59.1	87	48.1
HER2+	116	33.0	46	26.9	70	38.7
TN	48	13.6	24	14.0	24	13.3
Surgery type						
Mastectomy	303	86.1	142	83.0	161	89.0
Lumpectomy	49	13.9	29	17.0	20	11.0

*NST* neoadjuvant systemic therapy; *BMI* body-mass-index; *UOQ* upper outer quadrant; *LOQ* lower outer quadrant; *UIQ* upper inner quadrant; *LIQ* lower inner quadrant; *HR* hormone receptor; *HER2* human epidermal growth factor receptor 2; *TN* triple-negative

### Visualization, Success Rate, and Complications of IM-SLNB

Among the 352 recruited patients, 183 had IMSLN drainage identified by the intraoperative gamma probe. The IMSLN visualization rate was 71.9% (123/171) and 33.1% (60/181) among patients receiving initial surgery and NST respectively, and the difference was statistically significant (*P* < 0.001). In the group of patients receiving initial surgery, age (*P* = 0.044) and body mass index (*P* = 0.031) were negatively correlated with visualization of IMSLN. Clinical tumor stage, clinical nodal stage, tumor location, pathological type, histological grade, tumor subtype, and radiotracer intensity did not affect the frequency of IMSLN visualization (all *P* > 0.05). In the group of patients receiving NST, clinical tumor stage (*P* = 0.025) and pathological response of primary tumor (*P* = 0.047) were negatively correlated with visualization of IMSLN. Clinical nodal stage, tumor location, pathological type, pathological response of ALN, tumor subtype, and radiotracer intensity had no effect on IMSLN visualization rate (all *P* > 0.05).

All of the 183 patients underwent IM-SLNB successfully, and the time duration of IM-SLNB averaged 7 min (range 4–28 min per person). Intraoperative internal mammary vascular injury occurred in 3.8% of patients (7/183), but hemostasis was successful in all cases; a 1- to 5-mm pleural lesion was noted intraoperatively in 5.5% (10/183), and only one patient had a small (15%) apical pneumothorax seen postoperatively on chest x-rays that resolved without intervention.

### Distribution of IMSLN

**Fig. 2:**
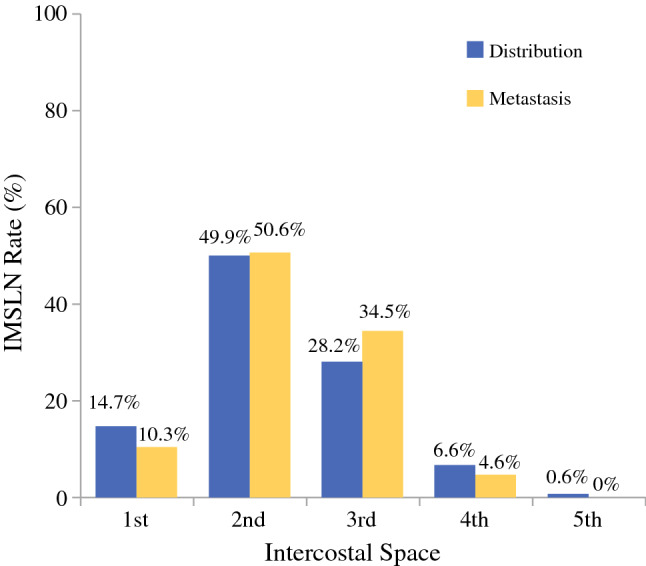
Distribution of IMSLN

IMSLN were dissected from the first intercostal space (ICS) to fifth ICS; the median number of IMSLN removed was two per person (total 347; range 1–7 IMSLN per person). The IMSLN were concentrated in the second ICS (49.9%) and third ICS (28.2%), followed by the first (14.7%), fourth (6.6%), and fifth (0.6%) ICS. Overall, 87 of the IMSLN were positive, of which 81 were H&E-positive and 6 were IHC-positive. There were 9 positive IMSLN in first ICS (10.3%), 44 in second ICS (50.6%), 30 in third ICS (34.5%), and 4 in fourth ICS (4.6%; Fig. 2).

### Metastasis of IMSLN

Initial Surgery Subgroup: The IMSLN metastasis rate was 39.8% (49/123) in the patients who underwent IM-SLNB (Table 2). Associations of clinicopathological factors with IMSLN metastasis were studied by univariate and multivariate logistic regression analyses: number of IMSLN, number of positive ALN, tumor size, and tumor location were significant independent predictors for IMSLN metastasis (all *P *< 0.05; Table 3).

**Table 2:** Characteristic correlation in initial surgery patients with IMSLN metastasis

Variables	IMSLN-positive *N *= 49		IMSLN-negative *N *= 74		*P*
	No.	%	No.	%	
Age					0.061
Median	51		47		
Range	34 ~ 70		33 ~ 72		
No. of IMSLN					**0.038**
Median	2		1.5		
Range	1 ~ 7		1 ~ 5		
No. of positive ALN					**0.000**
1–3	13	26.5	50	67.6	
4–9	22	44.9	20	27.0	
≥ 10	14	28.6	4	5.4	
Pathological T stage					**0.001**
T1	9	18.4	35	47.3	
T2	35	71.4	35	47.3	
T3	5	10.2	4	5.4	
Tumor location					**0.026**
UOQ	17	34.7	45	60.8	
LOQ	8	16.3	7	9.5	
UIQ	14	28.6	9	12.2	
LIQ	6	12.2	3	4.0	
Central	4	8.2	10	13.5	
Histological type					0.166
Ductal	45	91.8	72	97.3	
Lobular	1	2.0	1	1.4	
Micropapillary	3	6.1	1	1.4	
Histological grade					0.548
I	1	2.2	3	4.2	
II	25	55.6	42	58.3	
III	19	42.2	27	37.5	
Tumor subtype					0.116
HR +/HER2−	31	63.3	37	50.0	
HER2+	12	24.5	21	28.4	
TN	6	12.2	16	21.6	

Bold values inidcate statistically significant

*IMSLN* internal mammary sentinel lymph node; *ALN* axilla lymph node; *UOQ* upper outer quadrant; *LOQ* lower outer quadrant; *UIQ* upper inner quadrant; *LIQ* lower inner quadrant; *HR* hormone receptor; *HER2* human epidermal growth factor receptor 2; *TN* triple-negative

**Table 3:** Univariate and multivariate logistic regressions for prediction of IMSLN status

Variables	IMSLN status (positive vs. negative)			
	Univariate analysis		Multivariate analysis	
	OR (95% CI)	*P*	OR (95% CI)	*P*
No. of IMSLN	1.388 (1.007, 1.913)	**0.045**	1.579 (1.008, 2.475)	**0.046**
No. of positive ALN				
1–3	Reference		Reference	
4–9	4.231 (1.791, 9.995)	**0.001**	11.156 (3.136, 39.691)	**0.000**
≥ 10	13.462 (3.789, 47.825)	**0.000**	74.165 (6.941, 792.417)	**0.000**
Pathological T stage				
T1	Reference		Reference	
T2	3.889 (1.630, 9.278)	**0.002**	6.353 (1.751, 23.056)	**0.005**
T3	4.861 (1.079, 21.897)	**0.039**	9.000 (1.120, 72.353)	**0.039**
Tumor location				
UOQ	Reference		Reference	
LOQ	3.025 (0.951, 9.628)	0.061	9.734 (1.788, 52.984)	**0.008**
UIQ	4.118 (1.505, 11.263)	**0.006**	11.250 (2.222, 56.956)	**0.003**
LIQ	5.294 (1.188, 23.585)	**0.029**	3.724 (0.568, 24.430)	0.171
Central	1.059 (0.292, 3.834)	0.931	0.399 (0.051, 3.122)	0.382

Bold values inidcate statistically significant

*IMSLN* internal mammary sentinel lymph node; *OR* odds ratio; *CI* confidence interval; *ALN* axilla lymph node; *UOQ* upper outer quadrant; *LOQ* lower outer quadrant; *UIQ* upper inner quadrant; *LIQ* lower inner quadrant

NST Subgroup: Of the patients who underwent IM-SLNB, 52 patients had IMSLN-negative status (13 patients with Sataloff N-A and 39 with Sataloff N-B) and 8 patients had IMSLN metastases (5 patients with Sataloff N-C and 3 with Sataloff N-D), the metastasis rate of IMSLN after NST was 13.3% (8/60).

### Change in Staging and Therapeutic Recommendation

All of the 183 patients who underwent IM-SLNB successfully were evaluated to learn whether their staging changed with the additional information of IMLN. IM-SLNB led to a more accurate nodal category in all patients and to more advanced staging in IMSLN positive patients.

Initial Surgery Subgroup: Of the 49 IMSLN positive cases, 13 cases had 1–3 positive ALN and the nodal status changed from a pN1a to pN1c; 22 cases had 4–9 positive ALN and the nodal status changed from a pN2a to pN3b causing a anatomic stage migration from IIIA to IIIC; 14 cases had ≥ 10 positive ALN and the nodal status changed from a pN3a to pN3b. The details of pathological prognostic stage were as follows: IA in 3 patients, IB in 8 patients, IIB in 2 patients, IIIA in 15 patients, IIIB in 12 patients, and IIIC in 9 patients. Based on the unfavorable primary tumor characteristics and ALN metastasis, systemic treatment was indicated in all these patients and only two pN1 patients (1.6%) escalated their chemotherapy regimens due to positive IMSLN. However, more accurate of nodal staging in all these 123 IM-SLNB patients may have prompted modifications to the radiation strategy. In 49 IMSLN-positive patients, 13 patients had 1–3 positive ALN. The therapeutic strategy including IMLN radiation therapy (IMLN-RT) might be more important in these 13 patients as current guideline are mostly based on status of ALN.

**Fig. 3:**
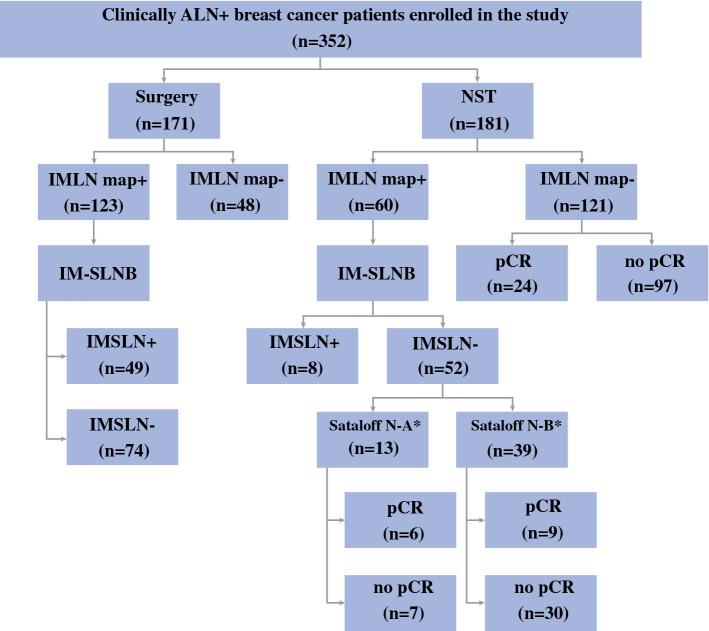
CONSORT flow diagram. *Sataloff scale of IMSLN (N–A: evidence of therapeutic effect, no metastatic disease. N–B: no nodal metastasis or therapeutic effect)

NST Subgroup: One of the 8 patients with IMSLN metastasis was not associated with ALN metastasis (ypN0 → ypN1b), 2 patients with 1–3 ALN metastases (ypN1a → ypN1c), 4 patients with 4–9 ALN metastases (ypN2a → ypN3b), and 1 case with ≥ 10 ALN metastases (ypN3a → ypN3b), and the postoperative anatomic stage also had been changed (0 stage → IB stage, IIA/IIIA stage → IIIC stage) (Fig. 3).

## Discussion

The IMLN is a common first-echelon nodal site for metastases and that IMLN metastasis is of prognostic significance in breast cancer. IMLN dissection was part of the standard surgical treatment in the 1950s and 1960s and is the main information source of IMLN metastasis to date. This radical surgical procedure was abandoned in the 1970s, because patient outcome studies showed that radical dissection did not improve survival at that time, when systemic therapy was not used.^10^ For most breast cancer patients, improvement of systemic therapy has significantly decreased the risk of death from distant metastasis, after which the optimized local therapy could, eventually, contribute more to improving survival in the era of molecular subtypes guided adjuvant/neoadjuvant systemic therapy. Therefore, the diagnosis and management of IMLN have attracted more and more attention in recent years.^1^,^2^,^11^

In recent years, the evidence supporting delivery of IMLN-RT in high-risk patients with IMLN metastasis is increasing,^12^^–^14 and NCCN Breast Cancer Clinical Practice Guidelines updated in 2016 has raised the level of IMLN-RT recommendation,^15^ IMLN-RT has attracted more and more attention to clinicians. With a median follow-up of 8.9 years, the DBCG-IMN (*N *= 3089) result showed that IMLN-RT could significantly improve OS (75.9% vs. 72.2%, HR 0.82, 95% CI 0.72–0.94, *P *= 0.005), reduce the mortality rate (20.9% vs. 23.4%, HR 0.85, 95% CI 0.73–0.98, *P *= 0.03), and the distant recurrence risk (27.4% vs. 29.7%, HR 0.89, 95% CI 0.78–1.01, *P *= 0.07).^16^ A meta-analysis of the three large clinical trials of EORTC 22922/10925 (*N *= 4004), MA 20 (*N *= 1832), and French trial (*N *= 1334) showed that radiotherapy of internal mammary and inner supraclavicular area for high-risk breast cancer patients (ALN positive and/or tumor located in central/inner quadrant of breast) could significantly improve OS (HR 0.85, 95% CI 0.75–0.96) (absolute benefit was 1.6%, 1.6%, 3.3% respectively), DFS (HR 0.85, 95% CI 0.77–0.94) and DMFS (HR 0.82, 95% CI 0.73–0.92).^17^ Subsequent to these results of the IMLN-RT trials, since 2016, NCCN Guidelines have updated recommendation of IMLN-RT for patients with ≥ 4 positive ALNs as category 1 and strongly consider IMLN-RT for patients with 1–3 positive ALNs (category 2A), both after mastectomy and lumpectomy.^15^

However, controversies persist regarding recommendation of IMLN-RT in all patients at high-risk of IMLN metastasis (ALN positive and/or medial tumor) even if the NCCN guideline has upgraded its recommendation. Cong et al. reviewed the extended radical mastectomy data and showed that the IMLN metastasis rate was 9.2% (4.4–16.8%), 19.6% (18.8–6.7%), and 38.3% (36.8–46.2%) for patients with 0, 1 ~ 3, and ≥ 4 positive ALN, respectively.^18^ Positive IMLN was found in approximately 9% patients with negative ALN and negative IMLN was found in approximately 60% of patients with ≥ 4 positive ALN. Therefore, current IMLN-RT indication (high-risk patients/no histopathological confirmation of IMLN) might result in over- and undertreatment, because high-risk does not represent IMLN metastasis and low-risk cannot exclude IMLN metastasis. Considering the short- and long-term cardiotoxicity of IMLN-RT, an effective and accurate method is needed to individualize the indication of IMLN-RT so that the benefit of IMLN-RT can be performed in patients with involvement of IMLN.^19^,^20^

Advances in sentinel lymph node biopsy of breast cancer enable IM-SLNB to provide a less invasive method for assessing IMLN than surgical dissection and may guide accurate nodal staging and individualized IMLN-RT.^9^ Although the 2009 American Joint Committee on Cancer incorporated the IM-SLNB concept, routine performance of the IM-SLNB in breast cancer patients remains a subject of debate due to the low IMSLN visualization rate and unclear clinical relevance. On the one hand, the internal mammary hotspots were only detected in a small proportion of patients (visualization rate 15%; range 13–37%) with traditional radiotracer injection technique, which has been the restriction for the IM-SLNB to date.^6^,^21^^–^23 In our earlier study, we tried injecting radiotracer with a modified technique (periareolar intraparenchymal, high volume, and ultrasound guidance) and got a high IMSLN visualization rate at 71.1%, which laid a technical feasibility for the further study and clinical application of IM-SLNB.^9^ On the other hand, previous studies of IM-SLNB showed that the IMSLN metastasis rate was only 8–15%, and most were accompanied by ALN metastasis. Only a small number of patients (2–8%) had positive IMSLN without ALN metastasis, which changed the lymph node staging from N0 to N + .^6^,^21^^–^23 Our center also reported a similar IMSLN metastasis rate of 8.1% in previous studies, of which five patients had lymph node staging changes from N0 to N + .^9^ In summary, based on the previous IM-SLNB indications (only in clinically ALN-negative patients), the clinical benefit of IM-SLNB was greatly reduced because of the low IMSLN metastasis rate. Therefore, the indications of IM-SLNB needs to be reconsidered.

Based on the experience of A-SLNB, the indication of SLNB (both axilla and internal mammary) are defined as clinically ALN-negative patient, which is adequate for axillary staging.^26^ However, the indication of IM-SLNB is still referred to axillary experience and is only performed in clinically ALN-negative patients, which is not adequate to current clinical practice. Actually, IMLN metastasis is mostly found concomitantly with ALN metastasis, Huang et al. retrospectively analyzed 2269 patients who received extended radical mastectomy and showed that the IMLN metastasis rate was 4.4%, 18.8%, 28.1%, and 41.5% for patients with 0, 1–3, 4–6, and ≥ 7 positive ALN, respectively.^7^ Based on the association of IMLN and ALN metastases, we tried to perform initial surgical treatment including IM-SLNB in clinically ALN-positive breast cancer patients in this study and found that 39.8% patients had IMSLN metastasis, which lead to accurate nodal stage and individual therapeutic strategy.

For patients receiving NST, the definition of nodal pCR just included the evaluation of ALN. Because IMLN metastasis has similar prognostic importance as that of ALN, the lymphatic metastasis and downstage should involve not only ALN but also IMLN. The definition of nodal pCR would not be completed without the pathology status of IMLN. It is necessary to perform IM-SLNB after NST to make clear of the whole nodal staging, because there were still 13.3% of patients with IMSLN metastases after NST. In addition, the IMSLN visualization rate was different statistically between initial surgery group and NST group (71.9% vs. 33.1%), and we speculated that the difference might be caused by the effect of NST on the lymphatic drainage pattern. Kuerer et al.^24^ reported that chemotherapy could alter the lymphatic drainage patterns by the shrinkage to and fibrosis of lymph vessels as well as by obstructing lymphatic channels with cellular material or tumor emboli. Li et al.^25^ also found that the lymphatic density around the breast tumor reduced significantly after NST, whereas the density of lymphatic vessels in the breast tumor was not significantly changed. However, researches on internal mammary lymphatic pathways after NST were limited, whether lymphatic vessels that drained into the IMLN were affected by NST has not been confirmed.

Another goal of this study was to determine whether there were independent predictors for IMSLN metastasis. Previous studies of IM-SLNB have demonstrated that age, tumor size, and ALN status were associated with the risk of IMSLN metastasis.^6^,^21^^–^23 However, these studies did not add much to the existing literature of the pattern of IMLN involvement, especially, histological/molecular subtypes were not found to be independently significant. In recent years, biological markers have been playing an increasingly important role in breast cancer staging and treatment decisions, and the concept of prognostic stage has been recommended in AJCC 8th edition. It could be found in our study that 11 patients (22.4%) had prognostic stage I disease despite the presence of ALN and IMLN metastasis. Although different histological/molecular subtype means different tumor recurrence patterns for all breast cancer patients, no correlation between histological/molecular subtypes and IMSLN metastasis was observed in our study. We speculated that this result might be related to the limitation of enrollment population (all the patients was clinically ALN-positive). In these patients, the risk of IMLN metastasis was mostly due to the influence of positive ALN, whereas the influence of histological/molecular subtypes would be relatively weakened.

## Conclusions

As a minimally invasive technique, we suggest that IM-SLNB should be routinely performed during mastectomy procedure, especially in clinically ALN-positive patients; and during lumpectomy procedure, because an additional 3-cm skin incision was required, IM-SLNB should be performed in clinically ALN-positive patients and selectively in clinically ALN-negative patients (IMLN high metastatic risk: positive ALN and medial tumor). These IM-SLNB indications above might help to make clear of the pathological nodal staging including both ALN and IMLN, improve the definition of nodal pCR, and potentially affect the therapeutic strategies. Our conclusion is driven by a growing understanding of individualized treatment strategies, which means that some patients will receive more locoregional therapy, whereas other females will receive less and still have very favorable results.

Finally, the deficiency of this study is to point out: in previous clinical practice of the axilla, a backup lymph node dissection had been performed before the clinical application to verify the accuracy of A-SLNB; the same goes for IM-SLNB: it also requires high level of validation study before clinical application in order to gain clinical acceptable accuracy and false-negative rates, which confirm that the IMSLN could accurately reflect the nodal status of internal mammary basin. Although the distribution of IMSLN in this study exactly coincides with the data of IMLN metastasis sites in previous extended radical mastectomy, a validation study involving IM-SLNB followed by IMLN dissection is still necessary for higher levels of evidence-based medicine. We are conducting a multi-center validation study, selecting ALN-positive breast cancer patients for IM-SLNB followed by 1–3 intercostal IMLN dissection, and current result (no public data) show that IMLN status could be evaluated accurately by IM-SLNB (false negative rate: 3.33%, 1/30). Furthermore, there are 41.4% (12/29) patients had additional, non-IMSLN metastasis in IMSLN-positive patients, hinting that IMLN-RT remained significant after the involved IMSLN was removed by IM-SLNB.
